# Supplementary material for: A Low-Cost Open Source Device for Cell Microencapsulation
Source: Materials (Basel). 2020 Nov 11;13(22):5090. doi: 10.3390/ma13225090 (PMC7696579; doi:10.3390/ma13225090)
Supplement: Supplementary file 1 [file materials-13-05090-s001.pdf]

materials

Supplementary Materials

A Low-Cost Open Source Device for Cell Microencapsulation

Miriam Salles Pereira [1,2], Liana Monteiro da Fonseca Cardoso [1], Tatiane Barreto da Silva [1], Ayla Josma Teixeira [1], Saul Eliahú Mizrahi [3], Gabriel Schonwandt Mendes Ferreira [3], Fabio Moyses Lins Dantas [3], Vinicius Cotta-de-Almeida [4,5] and Luiz Anastacio Alves [1,*]

[1] Laboratory of Cellular Communication, Oswaldo Cruz Institute, Oswaldo Cruz Foundation, 4365 Manguinhos, Rio de Janeiro 21045-900, Brazil; msmakeba@gmail.com (M.S.P.); lianamfc@gmail.com (L.M.d.F.C.); tatiane.barreto321@gmail.com (T.B.d.S.); aylajosma02@gmail.com (A.J.T.); alveslaa30@gmail.com (L.A.A)

[2] Volta Redonda University Center—UniFOA, Av. Paulo Erlei Alves Abrantes, 1325-Três Poços, Volta Redonda 27240-560, Brazil

[3] National Institute of Technology—INT, Rio de Janeiro Av. Venezuela, 82-Saúde, Rio de Janeiro 20081-312, Brazil; saul.mizrahi@int.gov.br (S.E.M.); gabriel.mendes@int.gov.br (G.S.M.F.); fabio.dantas@int.gov.br (F.M.L.D.)

[4] Laboratory on Thymus Research, Oswaldo Cruz Institute, Oswaldo Cruz Foundation, 4365 Manguinhos, Rio de Janeiro 21045-900,, Brazil; vca@ioc.fiocruz.br

[5] National Institute of Science and Technology on Neuroimmunomodulation (INCT-NIM), Oswaldo Cruz Institute, Oswaldo Cruz Foundation, 4365 Manguinhos, Rio de Janeiro 21045-900, Brazil; vca@ioc.fiocruz.br (VCA)

* Correspondence: alveslaa30@gmail.com; Tel.: +55-21-2562-1841; Fax: +55-21-25621816

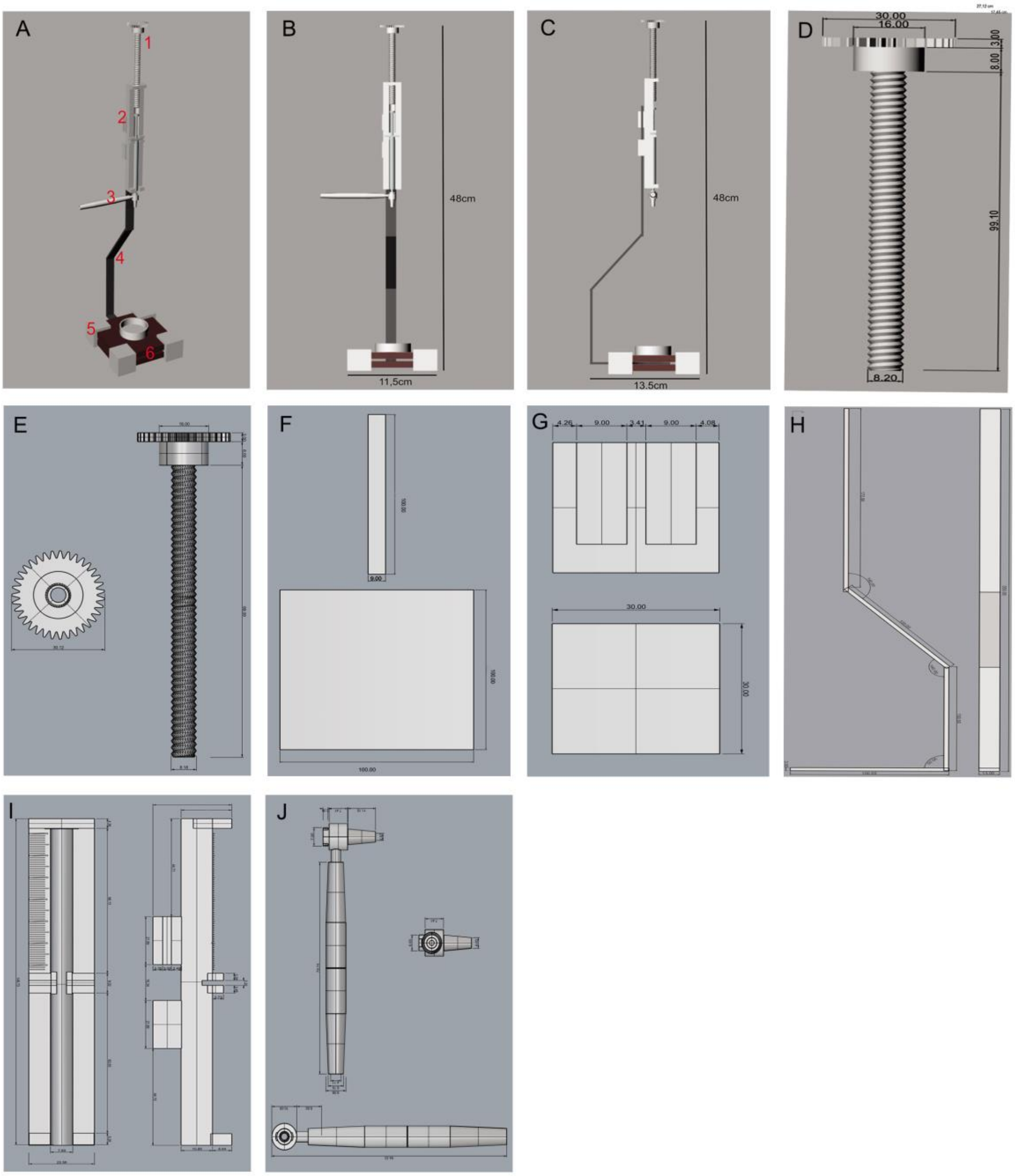

Figure S1. Cell encapsulation equipment produced in a 3D print. Measurements are displayed in millimetres (mm) or centimetres (cm).

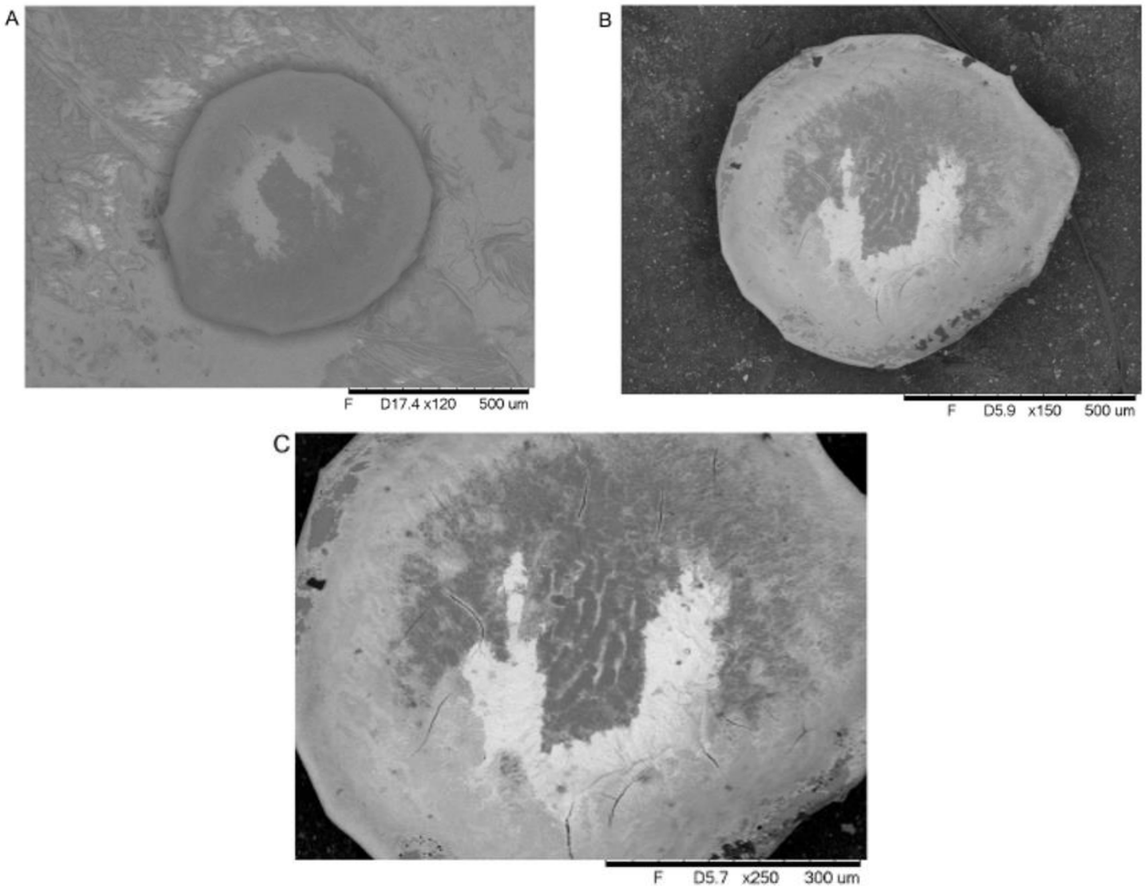

Figure S2. The sodium alginate microcapsules were evaluated by scanning electron microscopy-SEM (Hitachi TM 3000) fixed on carbon tapes at 5 kV, with magnification from 120 × (**A**), 150 × (**B**) and 250 × (**C**). Thus, was not possible to see the pores of the polymer.

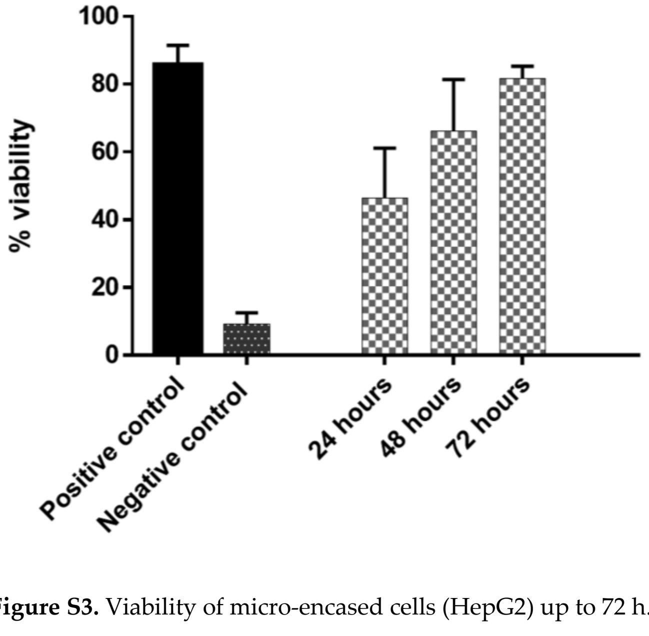

Figure S3. Viability of micro-encased cells (HepG2) up to 72 h.

© 2020 by the authors. Submitted for possible open access publication under the terms and conditions of the Creative Commons Attribution (CC BY) license (http://creativecommons.org/licenses/by/4.0/).
